# The Temporal Fine Structure of Background Noise Determines the Benefit of Bimodal Hearing for Recognizing Speech

**DOI:** 10.1007/s10162-020-00772-1

**Published:** 2020-10-26

**Authors:** H. C. Stronks, J. J. Briaire, J. H. M. Frijns

**Affiliations:** 1 Department of Otorhinolaryngology, Leiden University Medical Center, PO Box 9600, 2300 RC Leiden, The Netherlands; 2 Leiden Institute for Brain and Cognition, Leiden University, Leiden, The Netherlands

**Keywords:** cochlear implants, sensorineural hearing loss, hearing aids, speech intelligibility, speech perception, bimodal hearing

## Abstract

Cochlear implant (CI) users have more difficulty understanding speech in temporally modulated noise than in steady-state (SS) noise. This is thought to be caused by the limited low-frequency information that CIs provide, as well as by the envelope coding in CIs that discards the temporal fine structure (TFS). Contralateral amplification with a hearing aid, referred to as bimodal hearing, can potentially provide CI users with TFS cues to complement the envelope cues provided by the CI signal. In this study, we investigated whether the use of a CI alone provides access to only envelope cues and whether acoustic amplification can provide additional access to TFS cues. To this end, we evaluated speech recognition in bimodal listeners, using SS noise and two amplitude-modulated noise types, namely babble noise and amplitude-modulated steady-state (AMSS) noise. We hypothesized that speech recognition in noise depends on the envelope of the noise, but not on its TFS when listening with a CI. Secondly, we hypothesized that the amount of benefit gained by the addition of a contralateral hearing aid depends on both the envelope and TFS of the noise. The two amplitude-modulated noise types decreased speech recognition more effectively than SS noise. Against expectations, however, we found that babble noise decreased speech recognition more effectively than AMSS noise in the CI-only condition. Therefore, we rejected our hypothesis that TFS is not available to CI users. In line with expectations, we found that the bimodal benefit was highest in babble noise. However, there was no significant difference between the bimodal benefit obtained in SS and AMSS noise. Our results suggest that a CI alone can provide TFS cues and that bimodal benefits in noise depend on TFS, but not on the envelope of the noise.

## **INTRODUCTION**

Cochlear implants (CIs) are auditory prostheses offered to individuals who are severe-to-profoundly deaf. CIs can partly restore hearing by electrically stimulating auditory-nerve cells directly, thereby bypassing the degenerated hair cells in the cochlea. The performance of CIs has greatly improved since their inception by the advent of ongoing technological advances in stimulation strategies, processing power, and front-end processing. However, speech understanding of CI users in noisy environments remains a particularly challenging task. In fact, CI users are more susceptible to noise than normal-hearing listeners (Nelson et al. 2003; Zeng et al. 2005). Normal-hearing (NH) listeners perform better when exposed to amplitude-modulated noise by taking advantage of the silent periods in the noise (“listening in the gaps”), a phenomenon referred to as release from masking. Interestingly, CI users do not benefit from such silent gaps and, in fact, have more difficulty understanding speech in temporally modulated noise than in steady-state noise (Nelson et al. 2003; Qin and Oxenham 2003). Amplitude-modulated noise is especially detrimental to speech understanding for CI listeners when the temporal modulations match the rate of syllabic modulation (2–4 Hz) (Nelson et al. 2003). The sensitivity to temporally modulated noise is believed to be caused by the reduced access to pitch cues that are normally used to segregate speech and noise (Qin and Oxenham 2003). Pitch information is mainly restricted by the number of channels in the implant (Fu and Nogaki 2004) and by the way CIs process speech (Qin and Oxenham 2003; Hopkins and Moore 2009; Carroll et al. 2011).

Most speech processing strategies use only a restricted part of the acoustic frequency spectrum. Acoustic speech contains three different temporal components, namely envelope (fluctuations in overall amplitude within the frequency band of 2–50 Hz), periodicity (50–500 Hz), and fine structure information (600–10,000 Hz) (Rosen et al. 1992). Most speech processing strategies for CIs are based on continuous interleaved sampling (CIS) (Wilson et al. 1993; Wilson 2019). CIS-based strategies filter the incoming sound into a number of frequency bands equal to the number of electrodes. From each band, the amplitude envelope is extracted by low-pass filtering, typically with a cutoff frequency of 200–400 Hz (Wilson 2019). These envelopes are used to modulate the ongoing pulse carriers at the corresponding electrodes. The carrier rate is generally fixed at 1–2 kHz (Green et al. 2002). Notable examples of CIS-based strategies are spectral peak (SPEAK) and advanced combination encoder (ACE) developed by Cochlear Corp (Melbourne, Australia) (Kiefer et al. 2001; Skinner et al. 2002), High-Definition Continuous Interleaved Sampling (HDCIS) from Med-El (Innsbruck, Austria) (Dillon et al. 2016), and the HiRes strategies deployed in devices from Advanced Bionics (Valencia, CA) (Nogueira et al. 2009a). While these strategies differ in subtle ways, they all have in common that they retain the speech envelope, but discard the temporal fine structure (TFS). Given the cutoff frequency of the low-pass filter of speech processors, the extracted envelope usually contains both the speech envelope and a periodicity component, but no TFS. Further, the fixed pulse rates, as applied in most of the coding strategies, do not provide TFS cues, leaving only the temporal envelope to encode TFS (Carroll et al. 2011). The extracted envelope contains enough information to support speech recognition in quiet listening conditions (Shannon et al. 1995), yet it is not sufficient for speech understanding in noise (Fu et al. 1998), particularly when the noise is temporally modulated (Qin and Oxenham 2003; Stickney et al. 2004). TFS has indeed be shown to be an important cue for speech recognition in noisy environments (Swaminathan et al. 2016). Noise experienced in everyday life typically fluctuates, for instance the background music playing at a party, or competing talkers at the dinner table. Therefore, introducing TFS cues to improve speech understanding in temporally modulated noise has special relevance for CI users.

Advanced Bionics has attempted to introduce TFS in the HiRes 120 speech processing strategies by implementing the so-called spectral resolution (SpecRes) feature. After envelope extraction, SpecRes identifies the dominant spectral peak of each of the frequency bands. These spectral peaks are used to modulate the pulse rate of the carrier at the corresponding frequency to introduce TFS in the electric speech signal. Reportedly, HiRes 120 strategies are capable of representing TFS up to frequencies of approximately 2300 Hz (Nogueira et al. 2009a). The spectral peaks, however, are primarily used for current steering by modulating the current levels on two adjacent electrodes (Nogueira et al. 2009a; Wouters et al. 2015). As a consequence, no studies have explicitly assessed the benefit of TFS introduction by the SpecRes algorithm, as far as we are aware, but only the difference between HiRes and HiRes 120 processing on speech understanding, i.e., the combined effects of current steering and pulse rate modulation. These studies yielded inconclusive evidence for the benefits of HiRes 120 over HiRes, as they show small beneficial effects on some psychophysical tests, but not on other related tests (Firszt et al. 2009; Drennan et al. 2010; Donaldson et al. 2011; for a review, see Wouters et al. 2015).

Med-El has attempted to address the issue of TFS by introducing “fine structure processing” (FSP) that complements the conventional CIS strategy with TFS by introducing variable pulse rates on the apical electrodes. However, like SpecRes, the results obtained with FSP and related fine structure strategies have been mixed: while some studies found significant improvements of speech understanding in noise (Arnoldner et al. 2007; Riss et al. 2009; Vermeire et al. 2010; Müller et al. 2012), others did not (Magnusson 2011; Qi et al. 2012; Schatzer et al. 2015).

One other potential way to provide TFS and low-frequency information to CI users is to add a hearing aid (HA) in the contralateral ear (Qin and Oxenham 2003; Hopkins and Moore 2009; Oxenham and Simonson 2009). Contralateral amplification, or bimodal hearing, has been shown in a multitude of studies to improve speech understanding in noise (e.g., Armstrong et al. 1997; Dunn et al. 2005; Kong et al. 2005; Morera et al. 2005; Gifford et al. 2007; Dorman and Gifford 2010), although a minority of studies did not find a benefit, or even noted a disadvantage of wearing a contralateral HA (Mok et al. 2006; Liu et al. 2019). The fitting of a HA in the contralateral ear can be an attractive hearing solution for people with residual hearing in the contralateral ear, especially in countries where bilateral implantation is not the standard of care. However, people using a CI, and the hearing impaired in general, have a reduced ability to process TFS (Lorenzi et al. 2006; Moore 2008), which may limit the benefits of bimodal listening.

In the present study, we tested whether envelope-based speech coding strategies for CIs provide envelope cues only, as generally assumed, or whether there are TFS cues available to listeners using just their CI as well. Secondly, we investigated whether bimodal listeners can take advantage of acoustic TFS cues to aid in speech understanding in noise. We approached these research questions psychophysically by testing speech understanding in noise with and without contralateral amplification in a group of bimodal listeners (Table 1). We did not occlude the contralateral ear to make our observations more representative of the effects of fitting a unilateral CI user with a contralateral HA under everyday listening conditions.

**Table 1:** Subject demographics

ID	Age	HA	PTA_125–500_ (dB)	PTA_500–2000_ (dB)	HL (years)	CI (years)	CVC (%)	Etiology of hearing loss
S02	71	×	73	85	8	4.9	89	Possibly antibiotic-induced
S04	82	×	67	75	4	2.9	91	Possibly familial; progressive
S05	74	×	52	90	3	2.9	86	Familial; progressive
S06	62		53	60	9	4.4	100	Ménière’s disease; progressive
S07	86	×	50	80	8	3.3	97	Unknown; progressive
S09	86		77	65	4	2.4	78	Unknown; progressive
S10	67	×	73	65	4	3.9	76	Autosomal dominant (DFNA9)
S11	62	×	62	55	8	2.0	96	Autosomal dominant (DFNA9)
S12	75	×	60	65	7	1.7	80	Autosomal dominant (DFNA9)
S13	62		60	120	4	1.8	85	Unknown; possibly familial; progressive
S14	58		50	80	6	2.1	91	Congenital; familial; progressive
S15	79		62	120	6	1.5	83	Unknown; progressive
S16	85		45	110	3	1.7	87	Unknown; progressive
S17	78	×	60	70	5	1.5	92	Unknown; progressive
S18	50	×	67	70	5	1.5	86	Unknown; progressive
Total	Mean	Total	Mean	Median	Mean	Mean	Mean	
15	72	9	61	75	6	2.6	88	

*ID*: subject identification number; *HA*: subject was actively wearing a hearing aid when entering the study; PTA_125–500_: mean pure-tone audiometric threshold across 125, 250, and 500 Hz. *PTA*_*500–2000*_: median pure-tone audiometric threshold across 500, 1000, 2000 Hz. The median was used, because at 2000 Hz, some subjects had unmeasurable thresholds. In those cases, the technical maximum sound level + 10 dB was registered as threshold (110 + 10 = 120 dB). *HL*: duration of hearing loss (HL) defined according to Blamey et al. (2013), namely from the moment the subject was (almost) unable to use the phone (without lip-reading) up to the date of cochlear implantation date. If the date of inability to use the phone was unknown, the reported moment of profound hearing loss of the better hearing ear was used instead. *CI*: duration of cochlear implant use measured from the day of implantation. *CVC*: consonant-vowel-consonant score obtained in quiet at a 65-dB SPL speech level

**Fig. 1:**
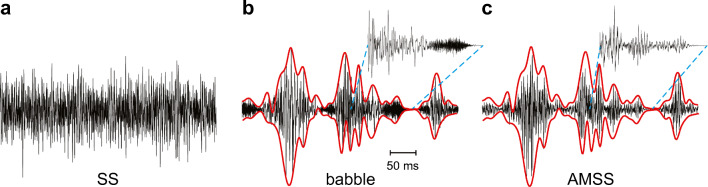
The three noise types used for speech-in-noise testing: steady-state (SS) noise (**a**), babble noise (**b**), and amplitude-modulated steady-state (AMSS) noise (**c**). AMSS was generated by modulating SS noise with the envelope of the babble noise (red lines). Insets show details of babble and AMSS noise

We tested three types of noise with different envelopes and TFS cues (Fig. 1 and Table 2). These noise types were steady-state (SS) noise, babble noise, and a combination of these two. SS noise has a “flat” envelope, i.e., it lacks the slow temporal amplitude modulations of speech, and is therefore also referred to as stationary noise (e.g., Lu and Cooke 2008; Luts et al. 2014). It is believed to mask speech peripherally by activating neural populations also involved in speech understanding. This peripheral masking has been referred to as “tonotopic masking” or “energetic masking” (Chatterjee 2003; Chatterjee and Oba 2004). SS noise was compared with two different amplitude-modulated noise types. Amplitude-modulated noise contains a temporally modulated, fluctuating envelope. It is considered to mask speech more centrally in the nervous system, as it does not require spectral overlap in the cochlea. The first amplitude-modulated noise type was a standardized male babble noise obtained from the International Collegium for Rehabilitative Audiology (ICRA) noise material (Dreschler et al. 2001). It was derived from English speech material, but virtually all intelligible speech components were removed by filtering. ICRA babble noise does not provide informational masking or harmonic structure, but temporal speech characteristics (TFS, envelope, and periodicity) are preserved. The second amplitude-modulated noise type was created by modulating the SS noise with the envelope of the babble noise to generate an amplitude-modulated steady-state (AMSS) noise.

**Table 2:** Characteristics of the stimuli used

Stimulus	Envelope	Spectrum	Spectral shape	Sex	*F*_0_ (Hz)	intelligible
SS noise	flat	LTASS-WN				
AMSS noise	AM	LTASS-WN				
Babble noise	AM	LTASS-WN (universal)	♂			
Speech	AM	Speech	♀	♀	215	✓

*SS*: steady-state; *AMSS*: amplitude-modulated steady-state; *AM*: amplitude modulated; *LTASS-WN*: long-term-average speech spectrum-shaped white noise; *♂/♀*: male/female; *F*_*0*_: fundamental frequency determined with “Praat” (Boersma and Van Heuven 2001). ICRA multi-talker babble is considered to be unintelligible, although individual single-talker babble may contain some residual speech information. The spectrum of the ICRA babble noise is universal LTASS (Byrne et al. 1994; Dreschler et al. 2001)

As TFS was expected to be inaccessible to CI listeners without contralateral amplification, our hypothesis was that speech recognition performance with a CI alone depends on the envelope of the stimulus, and not on its TFS. This was tested by evaluating speech recognition in different types of noise, using a closed-set testing paradigm for Dutch/Flemish speech stimuli. Noise stimuli were SS noise, babble noise, or AMSS noise. According to our hypothesis, speech recognition with a CI only should be better in the presence of SS noise than in amplitude-modulated noise, but it should not differ between babble and AMSS noise, as their envelopes are the same, and TFS is not a cue that CI processors convey to the listener.

As outlined above, a HA can potentially provide TFS cues by delivering low-frequency acoustic stimulation. TFS is especially important for speech recognition in amplitude-modulated noise. We hence hypothesized that bimodal benefits are larger for amplitude-modulated noise than in SS noise. On the basis of this hypothesis, our expectation was to find larger bimodal benefits in babble and AMSS noise than in SS noise. Since the SS and AMSS noise were speech-weighted (also referred to as speech-shaped noise), their overall spectrum resembled the female voice of the target speech (Luts et al. 2014). In contrast, the babble noise was a gender-filtered male noise (Dreschler et al. 2001) and this was expected to aid in the segregation of babble noise from the target speech in the bimodal condition. Hence, the bimodal benefit in AMSS noise was expected to be intermediate to SS and babble noise.

## **MATERIALS AND METHODS**

### Study Design and Subjects

This clinical trial was a prospective intervention study. The experimental design was unmasked (researcher and subject were both aware of the noise type and hearing configuration being tested), non-randomized (subjects were selected from the database of the institute according to the criteria listed below), and non-controlled (we used a cross-over design where subjects were their own control). Fifteen unilaterally implanted subjects (9 females, 6 males) with an Advanced Bionics system were included in this study. The demographics are listed in Table 1. All subjects were fitted with a research Naída Q90 processor, using their own speech processing strategy (HiRes Optima for all subjects) and their own threshold and maximal comfortable stimulation levels. The acoustic low-pass filter cutoff was 200 Hz. Inclusion criteria were (1) pure-tone audiometric thresholds of 80-dB HL or better at 125, 250, and 500 Hz in the non-implanted ear and (2) a CVC correct phoneme score of 80 % or better in quiet when using their CI alone. Based on their speech understanding in quiet, these listeners were regarded as average to above-average performers. Pre-operatively determined, unaided phoneme scores on the contralateral side obtained with headphones were 0 % at 65 dB for all subjects. Cognitive function was not an inclusion criterion. However, potential CI candidates receive an informal, psychological screening in our center to assess whether they are cognitively capable of completing the rehabilitation program.

**Fig. 2:**
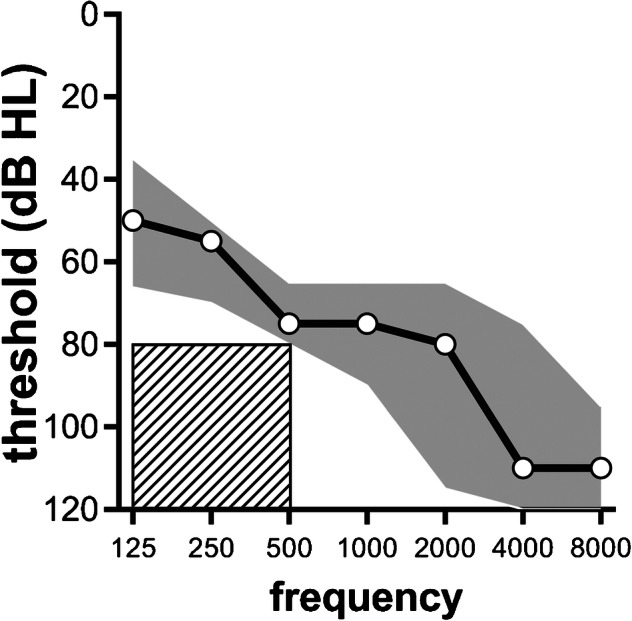
Median pure-tone audiogram for the non-implanted ear of all subjects. Shaded areas indicate the interquartile range. Hatched box: exclusion criterion based on residual hearing

All subjects had been fitted with a contralateral HA (Naída X UP, or Naída Link device; Phonak, Sonova Holding AG Stäfa, Switzerland) before testing and had been using it at home for at least 4 weeks. They used a bimodal fitting rule that emphasizes audibility of low-frequency sounds which carry TFS important for speech understanding in noise (Cuda et al. 2019). All subjects had used HAs before receiving a cochlear implant, and they used their own ear molds for this study, typically a full shell ear mold. In our center, unilateral CI wearers are often advised to stop wearing a contralateral HA at least temporarily after implantation to facilitate the rehabilitation process with the CI. While there are no peer-reviewed studies suggesting that discontinuation of the use of a HA facilitates rehabilitation following cochlear implantation, it is our center’s clinical philosophy to make this recommendation nonetheless. Eight subjects had participated in an earlier trial and had been fitted with a Naída X UP approximately 2 years before this study commenced and had been using that HA since (S02-S11). Two of these subjects had stopped using it in the period between the previous and current trials (S06, S09). Speech recognition of two subjects (S09, S10) had fallen a few percentage points below the criterion of 80 % on the CVC test during the intervening period, but they were included in this study nonetheless. Of the 7 newly recruited subjects, 2 already wore a Naída Link with the bimodal fitting rule (S12, S18) and 1 wore a Naída X UP device (S17). They had been fitted by their professional health care provider and wore the HA on a daily basis before being included. S17’s HA was re-fitted with the bimodal fitting rule, leaving the remaining settings intact. The other 4 subjects who had stopped wearing HAs after implantation altogether, and the two subjects who stopped using their HA after the preceding trial, were newly fitted with a Naída X UP or Naída Link device (S13–S16). These participants were encouraged to wear their HA daily for at least 4 weeks before being tested. The median, unaided pure-tone audiogram (with interquartile ranges) in the ear fitted with the HA is shown in Fig. 2. This study was approved by the Institutional Review Board of the Leiden University Medical Center, and adhered to the tenets of Helsinki (World Medical Association 2013). Informed consent was obtained from each subject.

### Speech Recognition Testing

Before testing, subjects were fitted with an experimental Naída Q90 CI speech processor and Naída Link HA. The Q90 processor was fitted with the subject’s own, preferred home-use threshold (T) levels and maximal comfortable (M) levels. The frequency range of the acoustic band-pass filter was set at “standard,” instead of “extended low,” which is currently the default setting. The “standard” filter option deploys a low-pass cutoff of 350 Hz (on electrode 1) and a high-pass cutoff of 8700 Hz (on electrode 16). The Naída Link HA was also fitted using the subject’s own, home-use settings. The primary outcome measure was the speech reception threshold (SRT), i.e., the signal-to-noise ratio (SNR) where a 50 % correct word score was obtained. To measure speech recognition, the Dutch/Flemish Matrix test (Luts et al. 2014) was deployed using the APEX 3 software platform (Francart et al. 2008a). The matrix test consists of sentences with a fixed syntax consisting of a name, verb, amount, color, and object. The words are drawn from a closed set of 50 words (10 names, 10 verbs etc.) voiced by a Flemish female speaker. An example of a translated sentence is “Emma has two black bicycles”. Each run consisted of the presentation of 20 sentences. Subjects repeated each sentence verbally, and the experimenter scored each correct word manually on a computer. Subjects were encouraged to repeat every word they heard, guessing was allowed, and no feedback was given. The speech level was adaptively varied using a staircase procedure, while the noise was presented at a constant level of 60 dBA in a diffuse field around the listener. The step size of the staircase was dynamically decreased after each reversal to a minimum of 0.1 dBA. The step size reduction depended on the number of reversals, as well as on the correct score obtained in the previous trial (Francart et al. 2008a). Typically, the speech level varied several decibels in the first few trials of the run, while in the later trials, when the speech level converged onto the 50 % word score, the variation was not more than 0.2 dBA. The SRT was determined by calculating the mean speech level across the last 6 trials.

The experiments were completed in 4 to 5 sessions of 2 to 2.5 h each. In each session, a single noise type (SS, babble, or AMSS noise) was tested. The first 4 sessions were used to measure SRTs in SS and babble noise. Noise type was alternated between sessions. The initial noise type was randomized. Hence, after 4 sessions, a test and a re-test were obtained for both noise types. In one subject, the re-test of the babble noise could not be performed, because the subject left the study for health reasons unrelated to hearing. AMSS was tested and re-tested in 11 subjects in a fifth session. In the remaining 3 subjects, these measurements could not be performed due to time constraints. Per session, at least 12 speech recognition tests were administered, each run lasting approximately 5 min. More tests were sometimes needed when tests were considered unreliable, e.g., when the SRT was not stable at the end of the run. During the first 4 sessions, additional data on noise reduction algorithms (beamformers and related algorithms) were obtained that are not presented here. The present data were obtained using omnidirectional microphone settings for CI and HA, and no beamforming or binaural processing was applied in the CI or HA. No other speech enhancement algorithms (e.g., ClearVoice or SoundRelax) were used. Only the processor microphone of the CI was used (i.e., no Tmic was used).

In each session, listening condition (CI only, HA only, and bimodal listening, i.e., CI + HA) was randomized. In the CI-only condition, the contralateral ear was not occluded. During the start of each session, the experience of the subject with the HA was informally assessed. We enquired whether they used the HA, how many hours per day they wore it, and what the advantages and disadvantages of the HA were, and we asked them to give the HA a single overall rating in terms of their overall satisfaction and experience with the HA as a complement to their CI, ranging from 0 (“it is a terrible experience”) to 10 (“the HA is a perfect add-on to my CI”). The daily overall use was confirmed through data logging from the HA.

The first two runs of a session were reserved for practice runs to minimize training effects during the session. During these practice runs, the subjects were encouraged to use a sheet with the 50 words included in the Dutch matrix test. For the first few subjects, the practice runs were both performed in the presence of noise. We noticed, however, that the subjects benefited more from doing the first run in quiet (to familiarize them with the speech material) and the second one in the presence of noise. The resulting SRT of the second trial minus 4 dB was used as the starting SNR in subsequent runs. During practice, subjects were listening bimodally.

### Noise Stimuli

**Fig. 3:**
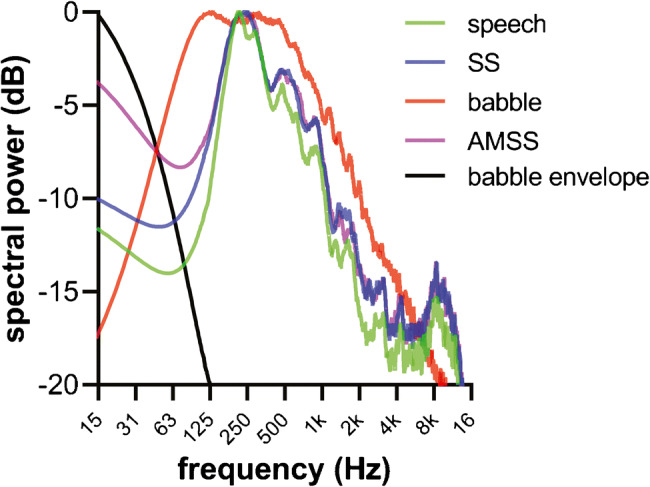
Frequency spectra determined by fast Fourier transformation of the speech material (green), steady-state (SS) noise (blue), babble noise (red), amplitude-modulated SS (AMSS) noise (purple), and the babble noise envelope (black)

Three noise types were used, namely steady-state (SS), babble, and amplitude-modulated steady-state (AMSS) noise. Sample waveforms can be found in Fig. 1, and their frequency spectra determined with fast Fourier transform are shown in Fig. 3. Table 2 lists their most important features, including the shape of the envelope and TFS characteristics.

The SS noise is included in the speech material of the Dutch/Flemish matrix test (Luts et al. 2014). It is an 11-s long noise fragment with a long-term average spectrum equal to the target speech. Note that the sound-pressure spectra show slight differences between the SS noise and the speech (Fig. 3), due to the fact that we ran the fast Fourier transform (FFT) on a selected number of sentences only. The SS noise file was randomly offset 7 times (varying from 1 to 7 s) to generate 8 uncorrelated noise sources. The offset was generated by cutting the initial part of the noise file and pasting it to the end. Each file was played on a different loudspeaker to generate a homogenous noise field around the listener.

The babble noise was generated using the 2-talker male babble from the ICRA noise files. The ICRA noise generation has been described in detail in Dreschler et al. (2001). In short, it was developed by sending a speech signal voiced by an English-speaking female through a band-split filter. Three filters were used, namely a low-pass filter (cutoff frequency 800 Hz), a band-pass filter (800–2400 Hz), and a high-pass filter (2400 Hz). Next, the sign of each sample in the 3 bands was randomly reversed with a probability of 0.5. This procedure effectively destroys the TFS, while preserving the envelope modulations in the three separate bands of the original speech signal (Dreschler et al. 2001; Holube 2011). ICRA multi-talker babble, as used in this study, is considered to be unintelligible (Dreschler et al. 2001). The bands were subsequently filtered using the same filter bank and then added. The phase was randomized by an FFT procedure to make the sound more pleasant, and the universal overall spectral shape of male speech was applied (Byrne et al. 1994). This male speech-filter had an attenuation slope of 12 dB/octave below 100 Hz and 9 dB/octave above 500 Hz (Fig. 3). Each of the two channels of the resulting 2-talker babble was a 300-s long sound fragment representing a single babble talker. Both channels were randomly offset three times (varying from 40 to 240 s), resulting in 8 uncorrelated files. Like the SS noise, each of the babble-noise files was played back individually on one of the 8 loudspeakers, generating a diffuse (but not homogeneous) field of babble noise.

The third noise type was a combination of SS and babble noise, referred to here as amplitude-modulated steady-state (AMSS) noise. It was generated by extracting the envelope from the babble noise by determining the magnitude of its analytic signal (Marple 1999). The resulting envelope had negligible power at 100 Hz or higher (Fig. 3) and was used to amplitude-modulate the SS noise, as shown in Fig. 1. Just like the babble-noise files, the AMSS files were randomly offset (40–240 s) such that they were presented in an uncorrelated manner via the loudspeakers. The spectrum of AMSS noise was nearly identical to that of SS noise and the speech material (Fig. 3). The only difference was a larger low-frequency component in AMSS noise due to the amplitude modulation by the envelope of the babble noise. Babble noise had a deviating spectrum. Most notably, it had a larger spectral power at low frequencies in the range of 50–250 Hz than SS and AMSS noise.

### Test Environment

**Fig. 4:**
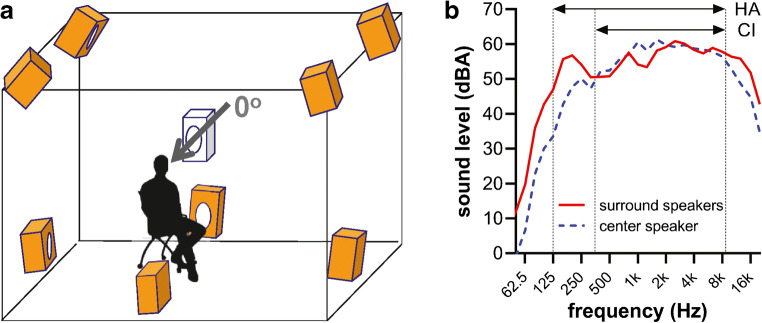
Test environment. Schematic of the homogeneous noise setup (**a**) and frequency characteristics of the loudspeakers (**b**). Speech was presented in front through a center loudspeaker and noise was presented through 8 loudspeakers positioned around the listener. The frequency characteristics shown in (**b**) were recorded in 1/3 octave bands of the surround loudspeakers (solid red line) used to present noise, and the center loudspeaker (dashed blue line) used for presenting the target speech stimuli. The stimulus was pink noise that was calibrated at the same overall sound level for the center and surround loudspeakers. Vertical lines: low-pass (350 Hz) and high-pass cutoff (8.7 kHz) of the CI speech processor (horizontal arrow labeled “CI”). The HA had an acoustic range (horizontal arrow labeled “HA”) with a lower low-pass cutoff (125 Hz), and a similar high-pass cutoff (Advanced Bionics LLC 2016)

Tests were performed in a sound-attenuated booth that measured 3.4 × 3.2 × 2.4 m (*l* × *w* × *h*). Noise was delivered through 8 surround loudspeakers (Control 1, JBL Corp., Los Angeles, CA) distributed symmetrically around the listener in 3D space (Fig. 4a) to yield a diffuse noise field when amplitude-modulated noise was applied, and a homogeneous field when SS noise was used. Four of the surround loudspeakers were positioned in the top corners of the room, the other 4 were placed in the middle of the side panels of the booth, approximately 40 cm above the floor. Each of the 8 loudspeakers was calibrated individually to yield an identical sound level as the other loudspeakers in the middle of the field, adding up to a noise level of 60 dBA where the subject’s head was located. The principle of creating a diffuse, homogeneous noise field as applied in this study has been described in more detail previously (Soede et al. 1993; Van der Beek et al. 2007). Speech stimuli were presented by a center loudspeaker (MSP5A monitor speaker, Yamaha Corp., Japan) placed 1 m away in front of the listener.

Because of space limitations, the surround loudspeakers were passive and smaller than the active center one. Hence their frequency characteristics differed slightly. The frequency spectrum of the center loudspeaker was characterized by generating SS noise through it and recording the long-term spectrum with a sound level meter (Rion NA-28, Rion Co. Ltd., Tokyo, Japan). The frequency characteristic of the surround loudspeakers was characterized similarly by generating uncorrelated SS noise through all of them and recording the frequency spectrum in the middle of the noise field (Fig. 4b). The reason all 8 speakers were recorded in the free field was because reflections also change the frequency characteristic. For instance, loudspeakers in the upper corners had a slightly different frequency characteristic than the ones near the floor due to different reverberation characteristics (individual frequency characteristics not shown). This also complicated the construction of a digital frequency filter to match the surround loudspeakers with the center one, because such a digital filter would need to be created for each loudspeaker individually. From Fig. 4b, it becomes apparent that the center loudspeaker had a higher energetic contribution at low and high frequencies, and a lower power in the mid-frequencies compared with the surround loudspeakers. This might have affected the masking efficiency of SS noise in particular. The digital sound file has the same long-term spectrum as the speech (Luts et al. 2014), but the actual acoustic noise was spectrally different from the speech due to the slightly different speaker characteristics, which likely decreased its energetic masking efficiency to some extent.

**Fig. 5:**
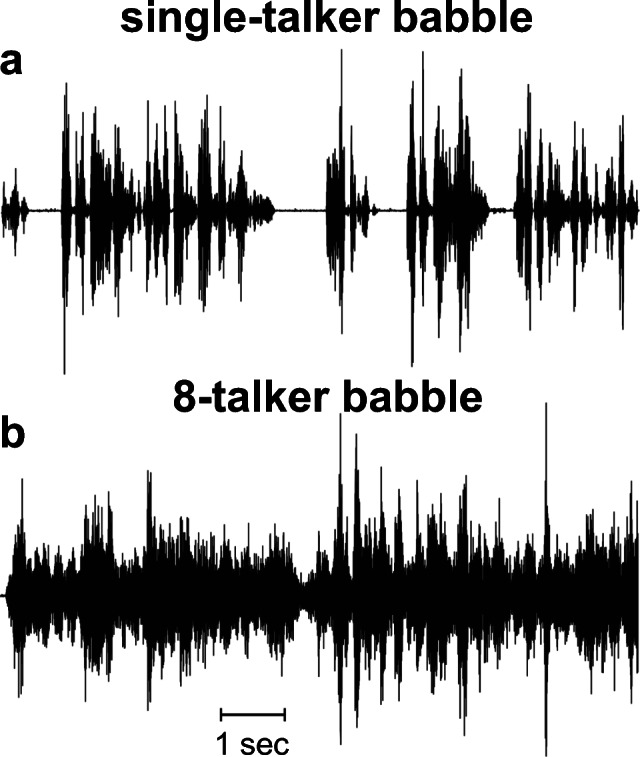
Effects of the 8-talker babble on modulation depth. 10-s fragments of the original ICRA babble noise (**a**) and the corresponding 8-talker babble noise as recorded from a CI and a KEMAR manikin (**b**) showing a reduction in the depth of amplitude modulation in the 8-talker condition

Calibration was performed using a sound level meter (Rion NA-28, Rion Co. Ltd., Tokyo, Japan) with the microphone positioned in the middle of the room at the position where normally the subject’s head would be. The SS noise was calibrated at 60 dBA by playing it over the 8 surround loudspeakers. The speech material was calibrated by playing the loudness-matched, speech-weighted SS noise over the center loudspeaker in front of the listener. The babble noise and AMSS noise were corrected for the overall sound level digitally by matching the root-mean-square value of the complete file length to that of the SS noise. Because there were 8 uncorrelated noise sources, the depth of amplitude modulation in the 8-talker babble and 8-“talker” AMSS in the diffuse noise setup was substantially reduced (Fig. 5). However, there was still appreciable fluctuation left, especially because of the varying speech-like sounds that fluctuated both temporally and spatially due to the 3D configuration of the loudspeakers in the room.

### Statistical Analysis

Statistical significance was tested using linear mixed modeling (LMM) using SPSS 23 for Windows (IBM Corp., Armonk, NY, USA). Two dependent variables were tested separately, namely SRTs and bimodal benefits. Bimodal benefits were calculated by subtracting the SRT obtained in the CI-only condition from the SRT obtained when listening bimodally. SRTs were used to investigate the effect of the three noise types on SRTs, i.e., to test whether amplitude-modulated noise decreases speech recognition more than SS noise. Bimodal benefits were used to investigate whether the benefit of bimodal hearing depended on noise type. Several fixed factors were included in the LMM, namely (1) noise type (N) to test the effects of TFS; (2) listening modality (CI + HA) to investigate the effect of bimodal hearing; and (3) prior HA use (HA_prior_). HA_prior_ was introduced, because 6 subjects included in this study had been using their HA for only 4 weeks after being recruited for this study. Four co-variates were accounted for in the model, namely two measures of residual hearing (PTA_125–500_ and PTA_500–2000_), performance with HA alone (SRT_HA_), and age. Better residual hearing may be correlated with higher bimodal benefit (Choi et al. 2016), although not all studies agree on this (Mok et al. 2006). Measures of residual hearing were the mean audiometric pure-tone thresholds at 500, 1000, and 2000 Hz (PTA_500–2000_) (Garretsen et al. 1997) and PTA_125–500_, reflecting the frequencies of our selection criterion, namely a threshold of at least 80 dB or better at each of these three frequencies. At 1000 and 2000 Hz, two subjects had audiometric thresholds that exceeded the output limit of the audiometer. In these instances, the maximum sound level of the audiometer was used and increased by 10 dB (115 + 10 dB at 1000 Hz, and 120 + 10 dB for 2000 Hz) to calculate PTA_500–2000_. The more frequently used PTA_500–4000_ was not used, because in 6 out of 15 subjects the threshold at 4000 Hz exceeded the maximum output level of the audiometer. Age was taken into account as it may impact a multitude of variables, ranging from speech understanding in noise to attention span and listening fatigue during testing (Lee 2015). Subject ID was included as a random variable. An intercept for the random and fixed variables was included in the LMM. The significance level was set at *α* = 0.05. The different fixed effects and co-variates were tested at this *α*, without correcting for multiple comparisons. No such correction was applied, because of the multitude of parameters analyzed in the LMMs, and the relatively small subject population, together resulting in a restricted power of this study. The number of subjects that met the inclusion criteria for this study was the most important limiting factor for subject recruitment in this study. The covariance matrix type of the fixed effects was set to “unstructured,” and that of the random subject-variable to “identity.” The variables PTA_125–500_ and prior HA use were examined in more detail by a standard regression analysis, or a 2-way repeated-measures ANOVA with prior HA use and noise type as within-variables, respectively.

## **RESULTS**

**Fig. 6:**
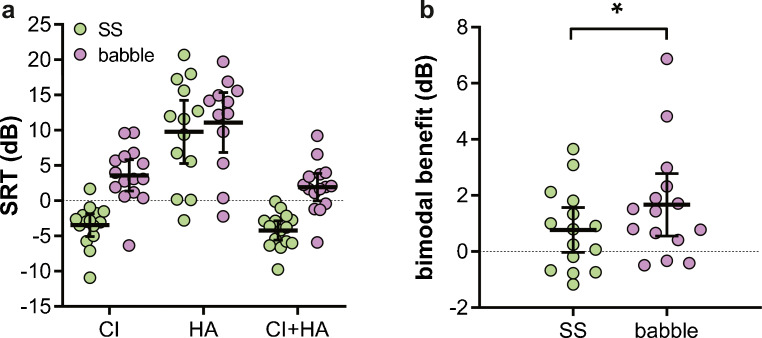
SRTs and bimodal benefits in 15 subjects. **a** SRTs were obtained in steady-state (SS) noise (green circles) and babble noise (purple circles) when listening with a cochlear implant only (CI), with a HA only (HA), and with both a CI and HA (CI + HA). Lower SRTs represent better speech recognition. **b** Corresponding bimodal benefits; higher values represent larger bimodal benefits. *P < 0.05

SRTs were obtained to compare speech recognition in the presence of SS and babble noise. An overview of the SRTs obtained with CI only (*N* = 15), HA only (*N* = 13 and 12 in SS and babble noise, respectively), and bimodal hearing (*N* = 15) is shown in Fig. 6a. Lower SRTs reflect better speech recognition scores in noise. SRTs with the HA alone could not be determined in 2 subjects in SS noise and in 3 subjects in babble noise, because of insufficient speech recognition with acoustic amplification alone. To test our hypothesis that speech recognition depends on noise type and on listening condition, we built an LMM. Babble noise resulted in a 6.5-dB larger deterioration of the SRT than SS noise (*P* < 10^−6^). Bimodal hearing improved the overall SRT by 1.3 dB (*P* < 0.0082). None of the other factors included in the LMM significantly affected SRTs. An overview of the model with its parameter estimates is shown in Table 3. Using the outcomes in the table, the LMM becomes:

**Table 3:** Fixed parameter estimates of the LMM with absolute SRTs as dependent variable

Parameter	LMM factor type	Estimate	SD	*t*	*P*
Intercept		− 21	7.4	2.8	0.021
CI + HA	Fixed factor	− 1.3	0.48	2.8	0.0081
N	Fixed factor	+ 6.5	0.50	13	< 10^−6^
HA_prior_	Fixed factor	+ 0.73	1.6	0.45	0.67
SRT_HA_	Fixed co-variate	+ 0.16	0.094	1.7	0.10
PTA_125–500_	Fixed co-variate	+ 0.13	0.079	1.6	0.14
Age	Fixed co-variate	+ 0.10	0.070	1.5	0.17

*CI + HA*: bimodal hearing (parameter estimate is 0 for CI only); *N*: babble noise (0 for steady-state noise); *SRT*_*HA*_: SRT with only HA; *PTA*_*125–500*_: average pure-tune audiometric threshold across 125, 250, and 500 Hz; *LMM*: linear mixed model

1 $$ SRT=-21-1.3\cdot \left[ CI+ HA\right]+6.5\cdot \left[N\right]+0.7\cdot \left[{HA}_{prior}\right]+0.2\cdot \left[{SRT}_{HA}\right]+0.1\cdot \left[{PTA}_{125-500}\right]-0.1\cdot \left[ Age\right] $$

where SRT is the predicted speech reception threshold; − 21 dB is the intercept; [CI + HA] is 1 when listening bimodally or 0 when listening with a CI only; [HA_prior_] is 1 if the subject wore a HA when recruited for the study or 0 when not; N (noise) is 1 for babble noise or 0 for SS noise; SRT_HA_ is the SRT when listening with a HA alone; PTA_125–500_ is the mean pure-tone audiogram across 125, 250, and 500 Hz; and age is the subject’s age. Suppose a bimodal listening subject of 65 years who was already using a HA when recruited in the study. This person had a PTA_125–500_ of 70 dB, was tested in babble noise, and had an SRT of + 15 dB with a HA alone. The predicted SRT then becomes: − 21 − 1.3 + 6.5 + 0.7 + (0.2 · 15) + (0.1 · 70) + (0.1·65) = + 1.4 dB. Removing the HA will yield a predicted SRT that is less favorable, namely + 2.7 dB.

In the second LMM, SRT differences (instead of SRTs as used above) were entered as the dependent variable (Fig. 6b). This second analysis allowed us to test our hypothesis whether the bimodal benefit differed between noise types. SRT differences were calculated by subtracting the SRT obtained when listening with a CI alone from the bimodal SRT. More negative values thus indicate a larger bimodal benefit. The LMM showed that the bimodal benefit was, on average, 1.0 dB larger in babble than in SS noise (*P* = 0.036). None of the other factors in the model had a significant effect on the bimodal benefit (Table 4). Parameter estimates are summarized in Table 4. The LMM becomes:

**Table 4:** Fixed parameter estimates of the LMM with bimodal benefit as dependent variable

Parameter	LMM factor type	Estimate	SD	*t*	*P*
Intercept		+ 4.1	4.5	0.91	0.39
N	Fixed factor	− 1.0	0.42	2.4	0.036
HA_Prior_	Fixed factor	− 1.5	1.0	1.5	0.18
SRT_HA_	Fixed co-variate	− 0.0018	0.063	0.029	0.98
PTA_125–500_	Fixed co-variate	− 0.062	0.049	1.3	0.24
Age	Fixed co-variate	+ 0.019	0.43	0.045	0.97

*N*: babble noise (parameter estimate is 0 for steady-state noise); *HA*_*prior*_: HA use when recruited for the study (parameter estimate = 0 when not wearing a contralateral HA); *PTA*_*125–500*_: average pure-tune audiometric threshold across 125, 250 and 500 Hz; *HA*: hearing aid; *SRT*_*HA*_: SRT with only HA; *LMM*: linear mixed model. Negative parameter estimates indicate a bimodal benefit

2 $$ B=+4.1-1.0\cdot \left[N\right]-1.5\cdot \left[{HA}_{prior}\right]-0.002\cdot \left[{SRT}_{HA}\right]-0.06\cdot \left[{PTA}_{125-500}\right]+0.02\cdot \left[\mathrm{Age}\right] $$

where *B* is the bimodal benefit (negative values indicating a benefit) and + 4.1 dB is the intercept. The remaining parameter symbols are equal to those in Eq. 1. Considering the same fictive subject as in the above example, the predicted benefit will become: + 4.1 − 1.0 − 1.5 − (0.002 · 15) − (0.06 · 70) − (0.02 · 65) = − 1.3 dB, i.e., a bimodal benefit of 1.3 dB. Switching to SS noise will yield a smaller benefit of 0.3 dB.

**Fig. 7:**
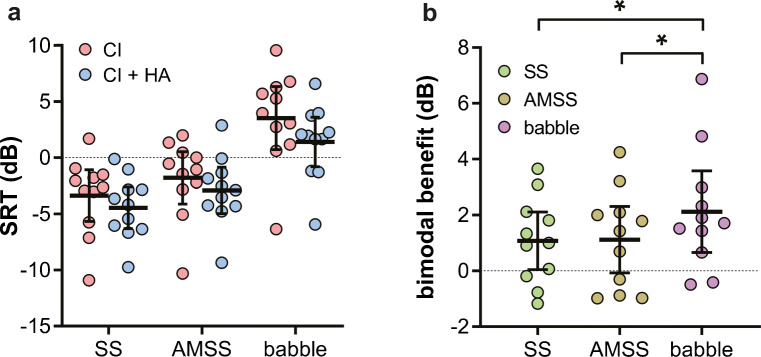
SRTs and bimodal benefits in 11 subjects. **a** SRTs were obtained in steady-state (SS) noise, amplitude-modulated steady-state (AMSS) noise, and babble noise with a cochlear implant only (CI, red circles) and with both a CI and HA (CI + HA, blue circles). Lower SRTs represent better speech recognition. **b** Corresponding bimodal benefits obtained in SS (green circles), AMSS (brown circles), and babble noise (purple circles). Higher values represent larger bimodal benefits. *P < 0.05

To investigate potential causes of the differences between the effects of SS and babble noise on speech recognition, we additionally determined SRTs using AMSS noise in 11 subjects. Figure 7a shows the SRTs obtained in all three noise types for this subpopulation. An LMM with the same parameters as Eq. 1 was used for significance testing. In this LMM, data from all 15 subjects were included for the SS and the babble noise, as an LMM corrects for the missing data in 4 subjects that were not tested in AMSS noise. The SRT obtained with the HA was omitted, as it was not obtained with AMSS noise. The babble noise resulted in the poorest (higher) SRTs (+ 6.6 dB relative to SS noise, *t* = 17, *P* < 10^−6^). AMSS also resulted in higher SRTs than SS noise (+ 1.6 dB, *P* = 0.00029), but less pronounced than the babble noise (*P* < 10^−6^). The bimodal benefits were examined including the subpopulation where AMSS was tested using the same parameters as in Eq. 2. Interestingly, the bimodal benefit (Fig. 7b) did not differ significantly between SS and AMSS noise (− 0.016 dB, *P* = 0.97), whereas in babble noise the benefit was significantly greater than in SS noise (− 0.90 dB, *P* = 0.036). From the 5 additional parameters entered in the four LMMs, namely PTA_125–500_, prior HA use, SRT with HA only, and age, none had a significant effect on the SRT or bimodal benefit (Tables 3, 4, 5, and 6).

**Table 5:** Fixed parameter estimates of the LMM with absolute SRTs as dependent variable

Parameter	LMM factor type	Estimate	SD	*t*	*P*
Intercept		− 18	6.7	2.6	0.023
CI + HA	Fixed factor	− 1.2	0.33	3.7	0.00050
N	Fixed factor	Babble: + 6.6	0.38	17	< 10^−6^
		AMSS: + 1.6	0.43	3.8	0.00029
HA_prior_	Fixed factor	− 0.0099	1.5	0.0066	1.0
PTA_125–500_	Fixed co-variate	+ 0.10	0.079	1.3	0.22
Age	Fixed co-variate	+ 0.11	0.066	1.7	0.11

*CI + HA*: bimodal hearing (parameter estimate is 0 for CI only); *N*: noise type (0 for steady-state noise); *HA*_*prior*_: HA use when recruited for the study (parameter estimate = 0 when not wearing a contralateral HA); *SRT*_*HA*_: SRT with only HA; *PTA*_*125–500*_: average pure-tune audiometric threshold across 125, 250 and 500 Hz; *LMM*: linear mixed model

**Table 6:** Fixed parameter estimates of the LMM with bimodal benefit as dependent variable

Parameter	LMM factor type	Estimate	SD	*t*	*P*
Intercept		3.6	3.2	1.1	0.28
N	Fixed factor	Babble: − 0.9	0.40	2.2	0.036
		AMSS: − 0.02	0.45	0.35	0.97
HA_prior_	Fixed factor	− 1.3	0.70	1.8	0.097
PTA_125–500_	Fixed co-variate	− 0.067	0.037	1.8	0.099
Age	Fixed co-variate	+ 0.0060	0.031	0.19	0.85

*CI + HA*: bimodal hearing (parameter estimate is 0 for CI only); *N*: noise type (0 for steady-state noise); *HA*_*prior*_: HA use when recruited for the study (parameter estimate = 0 when not wearing a contralateral HA); *SRT*_*HA*_: SRT with only HA; *PTA*_*125–500*_: average pure-tune audiometric threshold across 125, 250, and 500 Hz; *LMM*: linear mixed model. Negative parameter estimates indicate a bimodal benefit

**Fig. 8:**
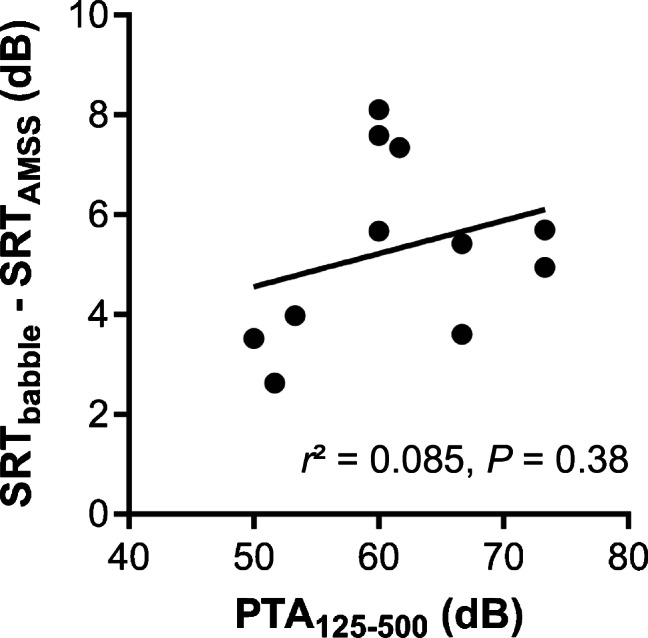
Difference SRT (SRT in babble noise (SRT_babble_) minus SRT in AMSS noise (SRT_AMSS_)) plotted against the average pure-tone threshold across the frequencies 125, 250, and 500 Hz (PTA_125–500_) in the CI-only condition. The linear correlation was not significant (F(1,9) = 0.84, *P* = 0.38)

Because we did not occlude the contralateral ear, the difference in SRTs between babble and AMSS noise in the CI-only condition might have been caused by the contralateral ear providing TFS cues. To examine this, the difference SRTs were plotted against PTA_125–500_ (Fig. 8). The difference SRTs were calculated by subtracting the SRT obtained in AMSS noise from the SRT obtained in babble noise. The correlation showed a weak positive trend (*r*^2^ = 0.085) between the two parameters that was not significant (F(1,9) = 0.84, *P* = 0.38).

To gain insight into the cause of the differences between the SRTs in the babble noise and AMSS, we visualized the TFS in the pulse-train output of the CI in the two noise types by running a simulation of the Advanced Bionics HiRes 120 speech processor kindly provided by Chen Chen (Advanced Bionics, LLC, Valencia, CA). The simulator accepted any audio wave file and processed it the same way as the speech processor would do (Nogueira et al. 2009b). It deployed a low-pass filter cutoff of 200 Hz. The envelope of the acoustic babble noise fragment that was used as input to the simulation corresponded with the envelope of the AMSS noise fragment. In other words, the acoustic envelopes were identical between the babble and AMSS noise, only the TFS and periodicity differed between the noise types.

**Fig. 9:**
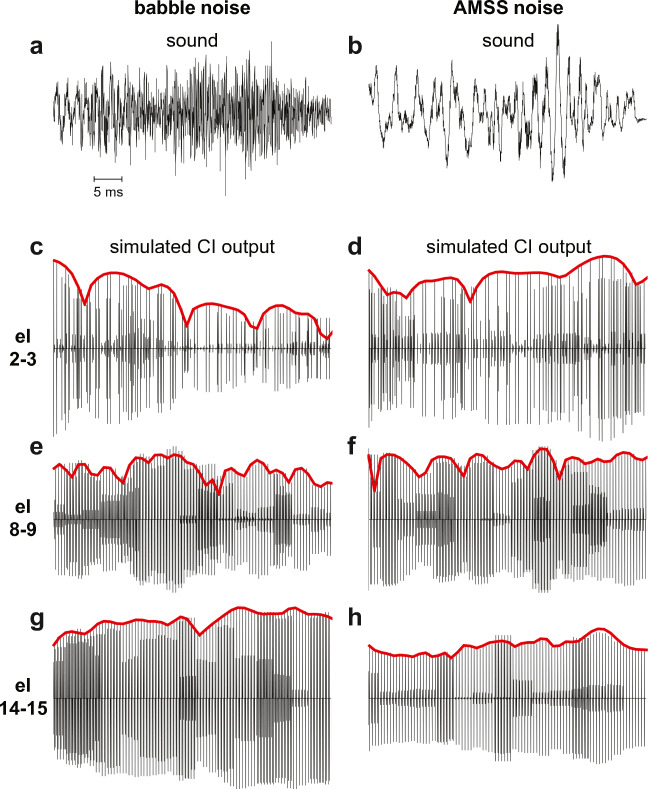
Computer-simulation of the CI pulse-train output to babble noise and amplitude-modulated steady-state (AMSS) noise. A short fragment of acoustic babble noise (**a**) and the corresponding AMSS noise (**b**) and their corresponding pulse-train outputs are shown for electrodes 2 and 3 (**c**, **d**), 8 and 9 (**e**, **f**), and 14–15 (**g**, **h**). The fragment resembled the consonant [s], with a prominent high-frequency component in it. The envelope of the babble noise was used to amplitude-modulate the steady-state state noise, yielding AMSS noise with a more prominent intermediate-frequency component. Electrodes in Advanced Bionics arrays are numbered from apical (1) to basal (16). Because of the current steering used in the HiRes strategies from Advanced Bionics, pulse trains are generated by activating pairs of electrodes

The simulated output shows the pulse train as generated by electrode pairs 2–3, 8–9, and 14–15 (Fig. 9). Outputs of electrode pairs are shown, as the HiRes 120 strategies, including the HiRes Optima strategy used by our study subjects, deploy current steering. In current steering, electrodes are activated pairs to steer the current to locations in-between electrodes (Advanced Bionics LLC 2009). The shown noise fragment in the babble noise trace (Fig. 9a) sounded like the consonant [s]. The corresponding fragment in AMSS noise (Fig. 9b) was perceptually reduced to a white noise burst. From the figure, it can be seen that there was a considerable difference in the timing and amplitude of the pulses between babble noise and AMSS noise (Figs. 9c-h), despite the fact that both shared the same acoustic envelope. In the AMSS noise, the TFS has been replaced by the TFS from the SS, which had a larger contribution of low frequencies in the acoustic noise fragment. It can be seen that the pulsatile output on the basally located electrode-pair 2–3 had a low-amplitude pulsatile output to the babble noise (Fig. 9c), corresponding to a smaller spectral low-frequency contribution. It had a higher-amplitude output to the AMSS noise (Fig. 9d), corresponding to the larger low-frequency component in the stimulus. Conversely, electrode-pair 14–15 located more in the base of the cochlea had a high-amplitude output to the babble noise (Fig. 9g), but a low-amplitude output to the AMSS noise (Fig. 9h).

The short, informal questionnaire taken at the start of each session revealed that 13 out of 15 subjects (87 %) always wore their HA at home (in combination with their CI). Two subjects (13 %) wore it for only a few hours per day. One of these two was the only person in the study population who preferred not to wear the HA. This person reported that the HA added nothing to the electric hearing but noise. This was not caused by little residual hearing, as the pure-tone thresholds were 25, 50, and 75 dB at 250, 500, and 500 Hz, respectively, all well within the study inclusion criterion. Contrary to this person’s subjective report, the SRTs actually improved by 0.2 dB and 0.8 dB in SS and babble noise, respectively. The one person who subjectively indicated to have worse speech understanding in noise with the bimodal fitting actually improved in both SS and babble noise by 2 dB and 5 dB, respectively. Fourteen out of 15 subjects (93 %) reported at least one benefit of the device, including improved sound quality, better speech understanding, and directional hearing during everyday use. 9 out of 15 reported disadvantages (60 %), including an increased hindrance caused by background noise, a negative impact on speech understanding in general, and outer ear discomfort due to the ear mold. The median rating, on a scale of 0 (HA is useless) to 10 (it is a perfect add-on to the CI) was 8.0. In Table 7 the three most frequently mentioned advantages and disadvantages are summarized.

**Table 7:** Subjective feedback on the HA

Advantages	*N* (%)	Disadvantages	*N* (%)
Improved sound quality (speech, music, overall “richness”)	10/15 (67 %)	Discomfort due to noise (echoes, whistling, background noise)	9/15 (60 %)
Improved speech understanding (in silence or noise)	9/15 (60 %)	Worse speech understanding (in silence or noise)	2/15 (13 %)
Binaural/directional hearing	7/15 (47 %)	Outer ear discomfort due to earpiece (buildup of ear wax, skin irritation)	2/15 (13 %)

## **DISCUSSION**

In line with expectations, we found that babble and AMSS noise were more detrimental to speech recognition than SS noise when subjects listened with a CI only. The envelopes of the babble and AMSS noise had envelopes that mimicked normal speech (Dreschler et al. 2001). Amplitude-modulated noise is thought to be more detrimental to speech recognition, as it effectively masks the envelope of the CI signal, especially when the envelope of the noise resembles that of syllabic modulation (Nelson et al. 2003). However, babble noise resulted in SRTs that were 5 dB higher than AMSS for both the CI-only and bimodal conditions. This finding contradicts the common assumption that CI users only have access to envelope cues (Wilson 2019), as the babble and AMSS noise had identical acoustic envelopes. These observations suggest that TFS information is made available via the CI alone. However, an alternative explanation for these results is the presence of periodicity cues in the speech and noise stimuli. The extracted envelope in the speech processor also contained periodicity cues (50–500 Hz (Rosen et al. 1992)), as the low-pass filter cutoff of the speech processor was set at 350 Hz in this study. Periodicity cues can be useful to extract *F*_0_ information, which in turn aids in segregating speech from background noise (Green et al. 2002; Hong and Turner 2009). The target speech in our study was voiced by a female talker with an *F*_0_ of approximately 220 Hz. The male babble noise did not contain an *F*_0_, but did have an overall male spectral shape (Table 2). In Fig. 3, it can be seen that for frequencies of 160 Hz and below, the levels of the babble noise exceeded those of the SS and AMSS noise. These differences are consistent with the male filter that was used to generate the babble noise (Dreschler et al. 2001), and the SS and AMSS noise types that had a long-term speech spectrum equal, or nearly equal, to the female target speech, respectively (Byrne et al. 1994). We, hence, cannot exclude that the higher susceptibility to babble noise in the CI-only condition was caused by periodicity cues due to these differences between the overall spectral shapes of the different noise types. However, if periodicity differences were an important cue, segregation of the female target speech should expectedly have been more efficient in the male babble noise than in AMSS noise. Since the reverse was true, we believe that the greater susceptibility of our subjects to babble noise in the CI-only condition was caused by acoustic properties other than periodicity.

The babble noise used in this study had time-varying spectral characteristics, such that it mimicked speech, including vocal effort (Dreschler et al. 2001). The SS and AMSS noise types lacked these spectro-temporal variations, as they were based on long-term average speech spectrum (LTASS) noise. LTASS noise is essentially filtered broad-band noise and lacks TFS. The envelope of the AMSS noise introduced temporal modulations to the LTASS noise, but it still lacked the TFS present in the babble noise. Hence, we believe that the speech-like characteristics of babble noise have resulted in a further reduction in speech recognition than observed when subjects were exposed to AMSS or SS noise. Of note is that the center loudspeaker producing the speech stimuli was from a different manufacturer than the surround loudspeakers, and their spectral characteristics differed from each other (Fig. 4b). As a consequence, the long-term spectrum of the LTASS noise did not exactly match that of the speech. This may have caused less-than-optimal masking of the target speech when listening in SS and AMSS noise.

Because we did not occlude the contralateral ear in the CI-only condition, subjects may have had some limited access to acoustic TFS cues from the contralateral ear, even without amplification. Therefore, the difference between the SRTs obtained in babble and AMSS noise in the CI-only condition might also have been caused by the unaided residual hearing in this ear. We did not, however, find a significant correlation between residual hearing and differences in the SRT between the two noise types in the CI-only condition. Because an absence of significance does not prove an absence of effect, we cannot exclude that the reduced speech recognition in babble noise relative to AMSS noise was due to the unaided contralateral ear. The bimodal benefits reported here may, hence, be conservative, as our subjects may have benefited from some acoustic hearing even without wearing a HA. In line with this notion, there was a trend towards larger bimodal benefits with less unaided residual hearing, i.e., better residual hearing resulted in lesser bimodal benefits. This trend was, however, not significant (Tables 4 and 6). We note that implantation criteria in The Netherlands are relatively stringent. As a consequence of this, the contralateral residual hearing is generally poor in CI users, as reflected by the audiograms in our study population (Fig. 2).

Besides the possible effect of unaided residual acoustic hearing, the apparent availability of TFS cues in the CI-only condition may be explained by the SpecRes feature implemented in the HiRes 120 speech coding strategies, including the HiRes Optima strategy used in the present study. However, as outlined in the Introduction, studies comparing the HiRes and HiRes 120 strategies have yielded mixed results. We were, hence, surprised to find sensitivity to TFS in our group of Advanced Bionics HiRes Optima users.

It has, however, been acknowledged by others that at least some TFS can be maintained in the CI output signal (Rubinstein and Turner 2003). Indeed, our simulation results do show substantial differences between the babble and corresponding noise fragments with the same acoustic envelope. We note that the simulation was based on the HiRes 120 strategy, while our subjects were fitted with the HiRes Optima strategy. At the time of this writing, no simulation model is available for the HiRes Optima. However, we do not expect this to affect any of our conclusions, as the HiRes Optima is just a more energy-efficient variant of HiRes 120 strategy, the only difference being that current steering is applied to half the inter-electrode distance instead of the full distance to conserve energy (De Jong et al. 2017). Our simulations will therefore have been affected only by slightly differing amplitudes of the pulses because of a different implementation of the current steering (Chen Chen, Advanced Bionics, Valencia, CA; personal communication).

In terms of the bimodal benefits, this study demonstrates that the addition of a contralateral hearing aid provides 1 dB more benefit in babble noise than in SS noise. This represents a substantial benefit, as speech intelligibility improves by more than 14 %/dB for the Flemish/Dutch Matrix test around the SRT (Luts et al. 2014), although the slope of the psychometric curve may be less steep in CI users (unpublished observations). The fact that we did not occlude the contralateral ear in the CI-only condition may have led to bimodal listening even in the absence of acoustic amplification with the HA. Hence, the average bimodal benefits reported here may be conservative estimates pertaining specifically to the addition of a HA. Given the restricted residual hearing in our population, as evidenced by a median PTA_500–2000_ of 75 dB and an SRT of approximately +10 dB on average, we assume that the benefits of acoustic hearing in the unaided condition was minimal.

The bimodal benefit in SS and AMSS noise was not significantly different. We expected a greater bimodal benefit for AMSS, however, given that TFS is particularly important for segregating speech in amplitude-modulated noise (e.g., Qin and Oxenham 2003; Turner et al. 2004; Hopkins and Moore 2009; Oxenham and Simonson 2009). These results indicate that the added bimodal benefit seen in babble noise was due to the TFS in the babble noise, and not to its envelope. In addition, the long-term frequency spectrum of the babble noise differed from those of SS and AMSS noise, and most notably by a larger contribution of low frequencies in babble noise, in particular in the frequency range of 50–250 Hz. We expect that the low-frequency cues provided by the HA were complementary to the CI, which delivers speech information dominated by high-frequency information. Technically, the frequency band of 50–500 Hz is considered to be periodicity and not TFS (Rosen et al. 1992). Hence, the HA might have delivered periodicity cues that might have contributed to the bimodal benefit observed in our study.

Most studies on the bimodal benefits in noise deployed either SS noise (e.g., Dunn et al. 2005; Mok et al. 2006), modulated noise (e.g., Pyschny et al. 2014; Dincer D’Alessandro et al. 2018); competing talkers (e.g., Kong et al. 2005; Gifford et al. 2007; Cullington and Zeng 2010; Pyschny et al. 2014), or babble noise (Tyler et al. 2002). These studies predominantly report benefits of bimodal hearing. However, none of them compared the bimodal benefit across noise types except one (Liu et al. 2019), as far as we are aware. In that study, the authors compared speech understanding in the presence of SS noise and of competing male or female talkers. They reported that bimodal benefits were not found for either noise type and that speech understanding was even worse when a male talker was masked with a male competing talker. These results are surprising, given the benefit of bimodal hearing generally reported in the literature, including the present study.

It has been shown by others that a HA can add acoustic fundamental frequency (*F*_0_) cues that are lacking in a CI (Qin and Oxenham 2003). *F*_0_ detection is important for speech understanding in noise and especially to differentiate between competing talkers (Kong et al. 2005). Early CI speech processing strategies were based on feature extraction that explicitly presented *F*_0_ and formant frequencies (Wilson and Dorman 2008), such as Multipeak (MPEAK) used in early Cochlear Corp. devices (Skinner et al. 1994). The noise stimuli used in the present study lacked harmonic structure and linguistic content. In this regard, we note that the magnitude of amplitude modulation of the noise in our setup was relatively low, because the noise was played through 8 uncorrelated channels. The addition of 8 uncorrelated noise sources effectively reduced the modulation depth (Fig. 5). Investigating the bimodal benefits in a single-talker babble noise or with competing talkers would therefore be informative to see whether the bimodal benefit would be further enhanced due to larger amplitude modulation and/or the presence of *F*_0_ cues.

Contralateral amplification can, theoretically, also offer binaural advantages that may increase speech recognition in noise. Binaural benefits can occur due to binaural redundancy, the head-shadow effect, and binaural squelch (Ching et al. 2005; Pyschny et al. 2014). Binaural redundancy manifests itself to the listener as signals being perceived louder when listened to with both ears (Potts et al. 2009). As a consequence, listeners are more sensitive to small intensity and frequency differences when listening binaurally, which aids in the separation of the target speech from noise (Ching et al. 2005; Avan et al. 2015). Binaural squelch, or binaural release of masking, is a centrally mediated segregation of a signal from noise. The signal and noise have to be presented at different angles such that temporal and intensity differences are produced at the two ears (Gray et al. 2009). However, the binaural squelch effect was expected to be small, because it is of little benefit to bimodal listeners (Francart et al. 2008b; Francart et al. 2009; Dorman et al. 2014; Kokkinakis and Pak 2014). The head-shadow effect is caused by the shielding effect of the head. If one ear is directed to the speech and the other to the noise, the former will have more favorable SNRs, as the noise is attenuated at the ear opposite to the noise source (Avan et al. 2015). In our setup, the SS noise field was homogeneous and the target speech was presented symmetrically from the front, and hence the head-shadow effect could not have played a role, since the SNR was the same in both ears. In contrast, for the amplitude-modulated noise types, the two ears may have experienced different SNRs, as the noise fluctuated and was uncorrelated across the different loudspeakers. However, given that AMSS did not show a larger bimodal benefit than the SS noise, we conclude that neither the head-shadow effect nor binaural squelch was a determining factor in the bimodal benefit. Hence, we reason that the larger benefit of contralateral amplification in babble noise may be mediated by the addition of low-frequency spectral information and TFS (Kong et al. 2005; Cullington and Zeng 2010; Oxenham and Kreft 2014), and possibly by binaural redundancy as well (Ching et al. 2005).

We note that AMSS was always tested in the last session, in which both test and re-test were performed. As the Matrix test has been associated with learning effects across sessions (unpublished observations on this group of subjects, and see De Jong et al. 2019), the SRTs may have been biased towards better scores for the AMSS. Learning effects, however, are unlikely to have affected bimodal benefits, as these were calculated as the difference between two SRT results and were hence corrected for any baseline performance shifts.

Apart from bimodal hearing and noise type, none of the other factors (PTA_125–500_, prior HA use, SRT with HA only, and age) had significant effects on the SRT or bimodal benefit (Tables 3, 4, 5, and 6) The lack of significant effect of PTA_125–500_ lends circumstantial support for the notion that wearing a contralateral hearing aid can be beneficial, even when the degree of hearing loss in the non-implanted ear is severe or profound (Ching et al. 2004; Ching 2005). The predominantly positive subjective feedback of the subjects in this study is in line with this view, as most of our study subjects had limited residual hearing.

## **CONCLUSION**

Our results support the notion that amplitude-modulated noise is particularly detrimental to speech recognition by CI users. In addition to temporal modulation, the TFS of the babble noise likely also affected speech recognition in the CI-only condition, which was an unexpected result, as it is generally assumed that TFS is not available after CIS-based speech coding. The current study also suggests that the benefit of bimodal hearing for speech recognition was greater in babble noise than in SS noise. This difference in benefit may have been dependent on the TFS cues present in the babble noise that were conveyed by the HA, and not on the amplitude-modulated character of the babble noise.
